# Using a non-invasive assessment of lung injury in a murine model of acute lung injury

**DOI:** 10.1136/bmjresp-2013-000014

**Published:** 2014-01-03

**Authors:** Siân Lax, Michael R Wilson, Masao Takata, David R Thickett

**Affiliations:** 1 Department of Clinical Respiratory Sciences, Centre for Translational Inflammation Research, University of Birmingham Research Laboratories, Queen Elizabeth Hospital, Birmingham, UK; 2 Department of Anaesthetics, Pain Medicine and Intensive Care, Faculty of Medicine, Imperial College London, Chelsea and Westminster Hospital, London, UK

**Keywords:** Pulmonary oedema, Respiratory Infection, Neutrophil Biology

## Abstract

Arterial oxygen saturation has not been assessed sequentially in conscious mice as a direct consequence of an in vivo murine model of acute lung injury. Here, we report daily changes in arterial oxygen saturation and other cardiopulmonary parameters by using infrared pulse oximetry following intratracheal lipopolysaccharide (IT-LPS) for up to 9 days, and following IT-phosphate buffered saline up to 72 h as a control. We show that arterial oxygen saturation decreases, with maximal decline at 96 h post IT-LPS. Blood oxygen levels negatively correlate with 7 of 10 quantitative markers of murine lung injury, including neutrophilia and interleukin-6 expression. This identifies infrared pulse oximetry as a method to non-invasively monitor arterial oxygen saturation following direct LPS instillations.

**Key messages**

- IT-LPS in mice causes a significant reduction in arterial oxygen saturation.
- Arterial oxygen saturation negatively correlates to markers of lung injury.
- Pulse oximetry can be used to define markers of injury that affect lung function.

## Introduction

Acute lung injury (ALI) and its most severe form, acute respiratory distress syndrome (ARDS), are defined in patients by acute onset, bilateral pulmonary infiltrations (reflecting pulmonary oedema) and hypoxemic respiratory failure (P : F ratio less than 300 mm (40 in SI units).^1–3^ Animal models are used to replicate pathological, physiological and histological changes in human ALI/ARDS.^4^

Lipopolysaccharide (LPS) is a potent activator of the innate immune system via toll-like receptor 4 pathways.^5^ Intratracheal (IT) LPS is a very reproducible technique which models many of the features in human ALI, typified by significant infiltration of neutrophils into the alveolar air spaces and expression of pulmonary inflammatory cytokines.^6–8^ Neutrophil accumulation post IT-LPS is followed by initiation of active resolution pathways which are required to inhibit neutrophil recruitment, and induce cell death and clearance.

The determination of murine lung injury following LPS typically involves assessment of cellular and cytokine responses which are correlated with markers of lung injury. Bronchoalveolar lavage (BAL) markers common to human and rodent lung injury include the protein permeability index (PPI; ratio of lung lavage fluid : plasma or serum protein levels) and the receptor for advanced glycation end products (RAGE).^9^ ^10^ BAL PPI and RAGE have been extensively used both in translational/murine studies as soluble markers of alveolar epithelial damage. However, in murine lung injury models repeated lung lavage is not practically feasible or ethically acceptable (in the UK). There is therefore a need for a non-invasive marker of lung damage that can be assessed sequentially in mice.

Pulse oximetry is widely used as an assessment tool for humans with acute and chronic respiratory conditions. The technical challenges of pulse oximetry in mice are high due to low pulse volume and very high heart rates. Recent advances in probe design and software analysis now make oximetry feasible as a non-invasive assessment of lung damage in murine models of lung injury. Pulse oximetry in murine studies is therefore increasingly popular as a technique to monitor the level of oxygen carried on arterial haemoglobin in conscious mice, without the use of surgery.^11–13^

In this study we used a pulse oximetry system to monitor lung function daily in a murine model of ALI. Our aims were first, to measure pulse oximetry in mice over the course of the inflammatory response following IT-LPS or phosphate buffered saline (PBS) as a control, and second, to compare oximetry readings to multiple lung injury and inflammation markers including PPI, RAGE, pulmonary neutrophils and local cytokine expression.

## Materials and methods

### Mice and IT instillations

Male 9–12-week-old wild type (WT) C57Bl/6 mice, with an average body weight of 25 g (±0.7 g) were obtained from Harlan UK Limited, Oxford, UK and maintained at BMSU, Birmingham University, UK. All experiments were performed in accordance with UK laws with approval of local ethics committees. IT instillations were performed as previously described.^14^ Briefly, mice were anaesthetised using intraperitoneal injections of metetomidine (60 mg/kg) and ketamine (10 mg/kg) and a fine polyethylene catheter (external diameter 0.61 mm and internal diameter 0.28 mm) passed into the trachea via the mouth under direct visualisation of the vocal cords. Fifty micrograms LPS (Source Biosciences, UK) in 50 µL sterile PBS or PBS alone were instilled. Mice were given 0.1 mL atipamezole to reverse the metetomidine and hydrated with two 0.5 mL saline subcutaneous injections, one immediately post IT and another 6 h later.

### Infrared pulse oximetry

Following IT instillations, the hair around the neck of each mouse was removed using Veet (Unilever, UK). Twenty-four hours post IT instillation and then every 24 h after that cardiopulmonary health status of each mouse was measured by MouseOx Plus (Starr Life Sciences Corp, USA) in accordance with manufacturer's instructions, up to and including 96 h (day 4) and also on day 9. Each mouse was very briefly anaesthetised using 5% isoflurane to facilitate placement of a CollarClip Sensor and allowed to acclimatise for 5 min. This time point was sufficient for animals to recover normal activities and physiological readings. Measurements were then recorded for 10 min. This time point was used to collect representative, error-free data due to the motion artefact.^15^ This was averaged for all parameters.

### Assessment of BAL fluid cellular and inflammatory markers

Mice were sacrificed by exsanguination, serum collected and BAL performed with two washes of 0.6 mL PBS/EDTA (2 mM). BAL fluid was centrifuged at 400 g at 4°C with supernatant aliquoted and either used directly or stored at −20°C for analysis of cellular and inflammatory markers. Markers of oedema and endothelial damage—PPI (BioRad protein assay)—epithelial damage—BAL RAGE (DuoSet ELISA, R&D systems, UK)—and inflammation—proinflammatory cytokines interleukin (IL)-6, IL-1β and tumor necrosis factors α (TNFα), neutrophil chemokines CXCL1/KC and macrophage-inflammatory protein-2 (MIP-2), and the epithelial repair growth factor vascular endothelial growth factor (VEGF; Fluorokine MAP Multiplex, R&D systems, UK)—were measured. These parameters were chosen because they are all well-characterised, quantitative markers of damage and inflammation used in several murine models of ALI/ARDS. The remaining cell pellets were analysed directly by flow cytometry.

### Flow cytometry

Cells pelleted from BAL fluid were assessed for neutrophil inflammation by flow cytometry using fluorophore-conjugated antibodies (eBioscience). Granulocytes were enumerated by gating on cells with a high forward and a high side scatter distribution. Neutrophils were defined as CD11c negative, CD11b^+^Gr1^hi^F4/80^−^ granulocytes. All flow cytometry data are presented per mL of BAL.

### Statistical analysis

All parameters were analysed using Prism 6 (GraphPad Software Inc, USA). Data were tested for normality and significance assessed by an ordinary one-way analysis of variance. Two-tailed Student t tests were also used as indicated in the text. Linear regression was calculated by two-tailed Pearson correlations. All data are expressed as the mean of three experiments, each with at least three mice/time point (±SE of the mean).

## Results

### IT-LPS but not PBS causes a significant decline in lung function

**Figure 1:**
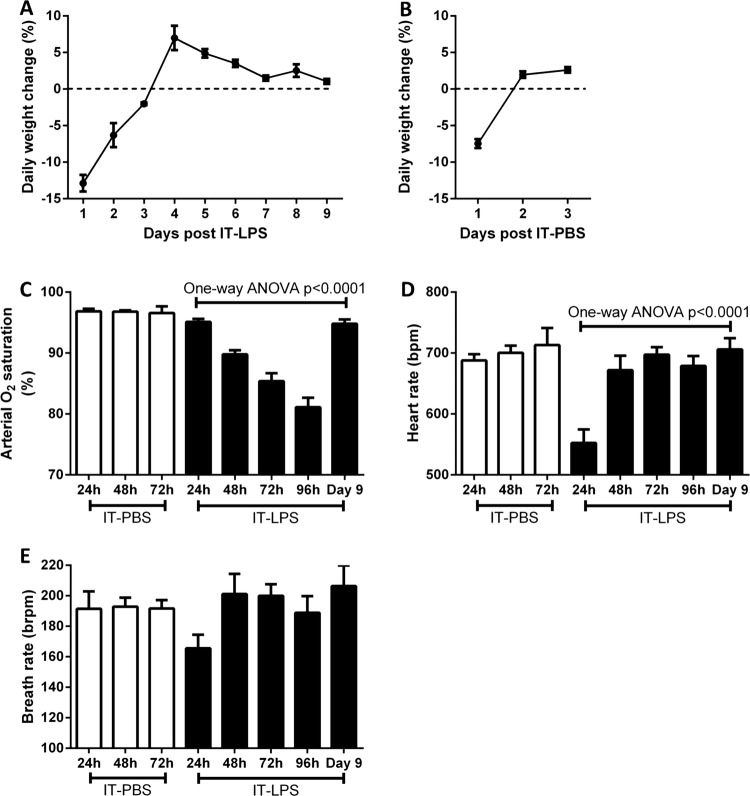
Weight and cardiopulmonary parameter changes in C57Bl/6 mice post IT-LPS or PBS. Weight changes were assessed in C57Bl/6 instilled via IT route with 50 µg LPS (A) or 50 µL PBS (B). Arterial oxygen saturation (C), heart rate (D) and breath rate (E) were monitored using infrared pulse oximetry following IT-PBS as a control (white bars) compared to IT-LPS instilled mice (black bars). IT-LPS, intratracheal lipopolysaccharide; PBS, phosphate buffered saline.

Following IT-LPS, the general health status of the mice was assessed daily by measuring weight loss of the animals. By 96 h (day 4) post-IT instillation, the mice started to gain weight indicating an improvement in health status (figure 1A). To control for the effects operation and LPS challenge a separate group of mice were instilled with PBS via IT injection. Weight loss was only observed 24 h post IT-PBS, which is likely due to the use of intraperitoneal anaesthetic which reduces the mouse's water and food intake within the first 24 h (figure 1B). To monitor the physiological consequence of causing ALI in mice we used infrared pulse oximetry on conscious, non-anaesthetised, mice. In mice given PBS instillation arterial oxygen saturation (SaO_2_; p=0.9621), heart rate (p=0.7025) and breath rate (p=0.9875) did not significantly alter during 72 h post IT. A reduction in SaO_2_ was observed from 48 h post IT-LPS (figure 1C). By 96 h post IT-LPS, mean saturation had reduced further to 81.1% (±1.6%). Oxygen saturation normalised by day 9, confirming that IT-LPS-dependent lung injury causes a significant but recoverable decline in lung function. In contrast, breath rate dropped only at 24 h post IT-LPS and then returned to levels similar to PBS controls (figure 1D). This cardiac suppression observed at 24 h post IT-LPS has been appreciated for LPS-based mouse models previously.^16^ Breath rate of LPS-treated mice did not alter significantly during the time course (p=0.0926; figure 1E).

### IT-LPS results in significant cellular inflammation and local cytokine release

**Figure 2:**
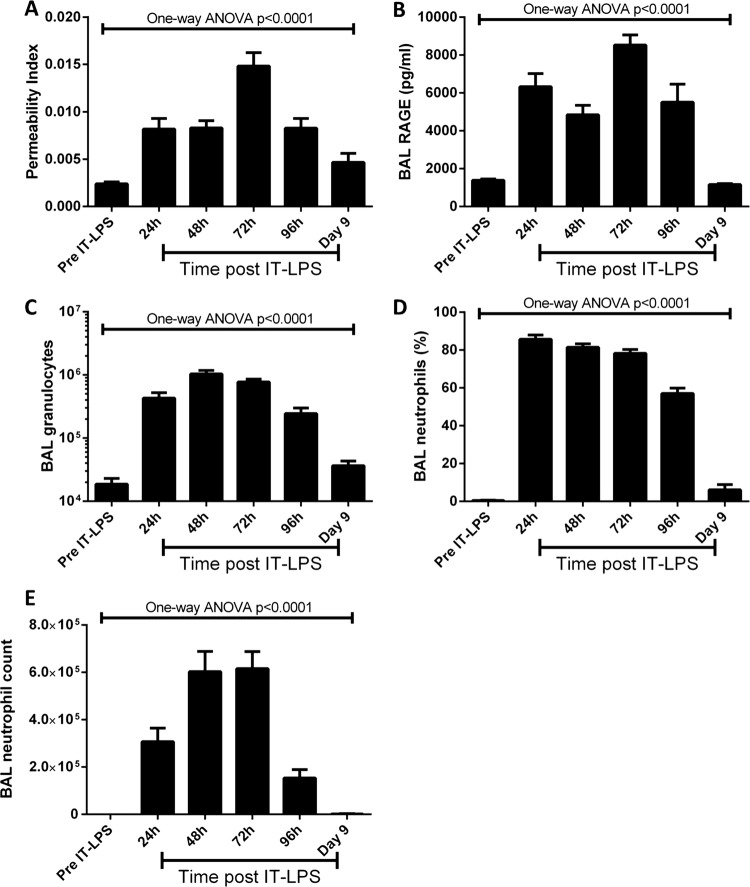
Markers of lung injury in C57Bl/6 mice post IT-LPS. C57Bl/6 mice were instilled via IT route with 50 µg LPS. Markers of endothelial barrier permeability (A) and alveolar epithelial cell damage by assessing RAGE expression (B) were assessed daily. The total number of granulocytes in BAL fluid was also enumerated per mL (C). The percentage (D) and number (E) of neutrophils were analysed using flow cytometry. ANOVA, analysis of variance; BAL, bronchoalveolar lavage; IT-LPS, intratracheal lipopolysaccharide; RAGE, receptor for advanced glycation end.

We analysed lung damage, cellular infiltration and inflammatory cytokine responses of WT mice to IT-LPS every day for 4 days and at 9 days post IT-LPS. IT-LPS caused significant lung injury as assessed by PPI and alveolar epithelial cell damage (BAL RAGE expression; figure 2A,B, respectively). Both parameters peaked at 72 h post IT-LPS from which they decreased. PPI levels at day 9 post IT-LPS remained significantly higher compared to preinstillation controls (0.0024±0.0002 vs 0.0047±0.0009, p=0.0030).

IT-LPS results in significant pulmonary granulocyte infiltration 24 h post IT-LPS which peaked at 48 h (figure 2C). As expected, cellular infiltration consisted primarily of neutrophils, with percentage peaking 24 h post IT-LPS (figure 2D). Although the numbers of BAL neutrophils are significantly reduced by 96 h post IT, BAL neutrophilia remained significantly elevated 9 days post LPS compared to resting levels (109±50 vs 1994±764, p=0.0009; figure 2E).

**Figure 3:**
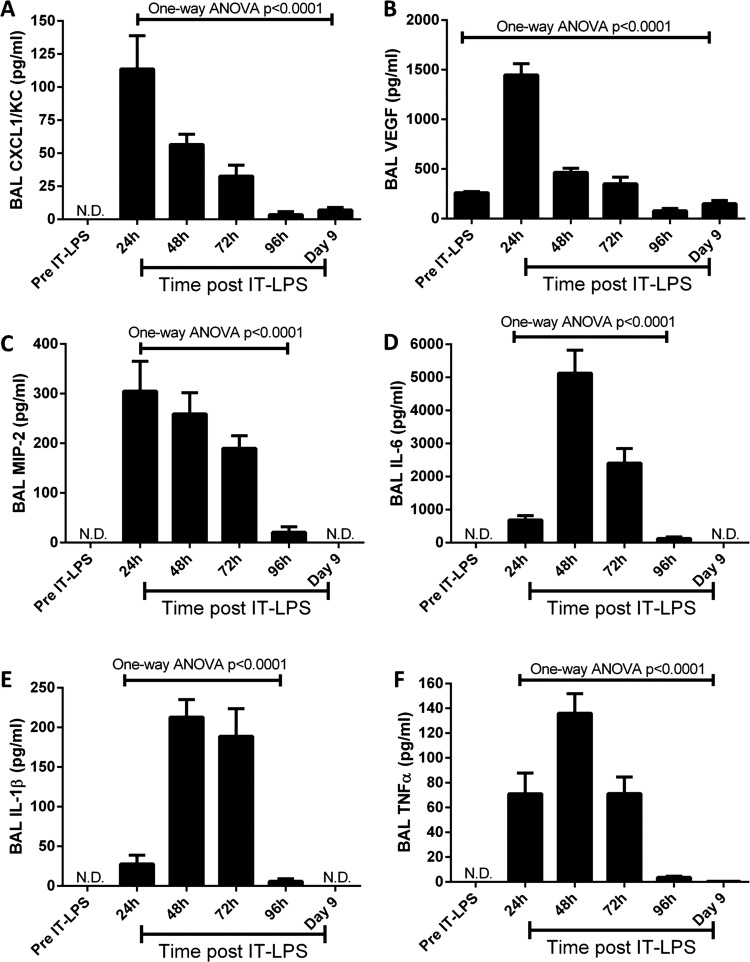
Pulmonary cytokine expression in C57Bl/6 mice post IT-LPS. C57Bl/6 mice were instilled via IT route with 50 µg LPS. BAL fluid was collected daily following IT-LPS and the expression of inflammatory cytokines assessed; CXCL1/KC (A), VEGF (B), MIP-2 (C), IL-6 (D), IL-1β (E) and TNFα (F). ANOVA, analysis of variance; BAL, bronchoalveolar lavage; IL, interleukin; IT-LPS, intratracheal lipopolysaccharide; MIP-2, macrophage-inflammatory protein-2; N.D., not detected; TNFα, tumor necrosis factors α; VEGF, vascular endothelial growth factor.

Expression of well-characterised inflammatory cytokines and chemokines was also analysed. Levels in BAL fluid peaked at either 24 h (CXCL1/KC, VEGF and MIP-2) or 48 h (IL-6, IL-1β and TNFα) post IT-LPS (figure 3A–F). Some subtle differences were observed during resolution; while CXCL1/KC, IL-1β and TNFα expression resolved by 96 h (with measured levels below the sensitivity of the assay), MIP-2 and IL-6 remained elevated (20.1 and 127.3 pg/mL, respectively) at 96 h and only by 9 days post IT-LPS did levels return to those of resting mice (figure 3C,D, respectively). Moreover, while VEGF resolved to pre-IT-LPS levels by around 72 h post IT-LPS (261.0±12.0 pg/mL), expression continued to fall below those of resting mice at 96 h (79.8±22.4 pg/mL, p<0.0001) and by day 9 levels still remained lower than those observed pre-IT instillations (151.3±32.0 pg/mL, p=0.0083; figure 3B). These changes in VEGF reflect the pattern, albeit with a different time course, seen in human ALI and have not been reported previously.

### IT-PBS induces mild cellular inflammation without affecting lung permeability

**Figure 4:**
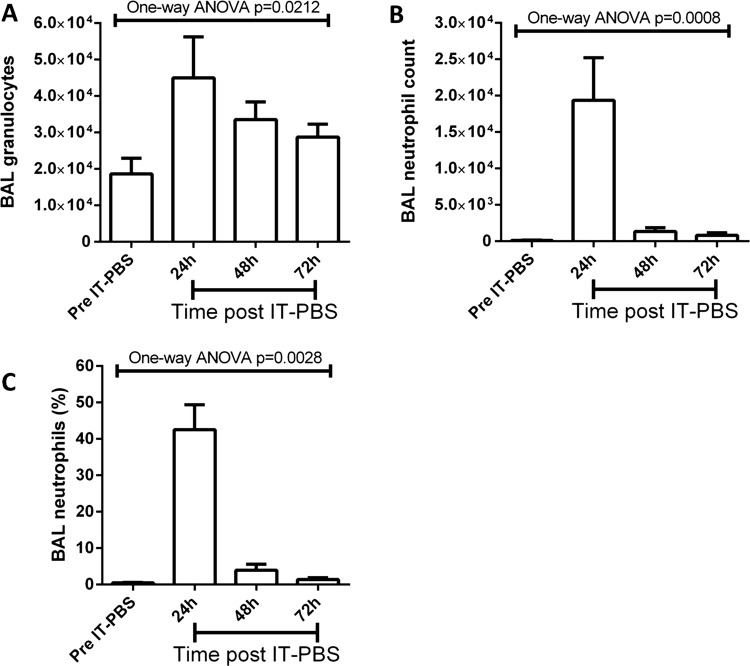
Granulocytic pulmonary infiltrates in C57Bl/6 mice post IT-PBS. C57Bl/6 mice were instilled via IT route with 50 µL PBS. BAL fluid was collected daily and pulmonary granulocytic infiltrates (A) and neutrophilia (B and C) assessed. ANOVA, analysis of variance; BAL, bronchoalveolar lavage; IT, intratracheal; PBS, phosphate buffered saline.

As a control, a second group of mice were instilled with IT-PBS and indexes of pulmonary damage, cellular infiltration and cytokine expression measured for 3 days. No significant changes in PPI or RAGE expression were observed at all time points measured following IT-PBS (data not shown). A small but significant granulocytic infiltration was observed 24 h post IT-PBS which resolved within 72 h (figure 4A). Cellular infiltrates contained neutrophils, however, these were at a much lower proportion than IT-LPS instilled mice (figure 4B,C). Consistent with no epithelial cell damage, expression of inflammatory cytokines was not observed above the detection threshold of the assays performed again validating the use of PBS as a non-inflammatory control substance (data not shown).

### SaO_2_ correlates to indices of lung injury

Having shown that cardiopulmonary parameters can be measured to assess lung function following IT-LPS we investigated how these parameters relate to quantitative markers of lung inflammation and damage. Table 1 displays the R^2^ value and p value of each cardiopulmonary parameter correlated to the lung injury, inflammatory cell recruitment and cytokine expression data—significant data are highlighted in bold. SaO_2_ negatively correlates with most inflammation markers measured (7 of 10).

**Table 1:** Correlations of markers of lung injury and inflammation, with cardiopulmonary parameters measured in C57Bl/6 mice post IT-LPS and PBS

Lung injury marker	Arterial oxygen saturation		Breath rate		Heart rate	
	R^2^	p Value	R^2^	p Value	R^2^	p Value
Permeability index	**−0.154**	**0.0122**	−0.075	0.0916	**−0.291**	**0.0004**
RAGE expression	**−0.219**	**0.0282**	−0.002	0.8247	−0.077	0.1619
Granulocyte count	**−0.108**	**0.0408**	0.044	0.1792	**−0.136**	**0.0150**
Neutrophil count	**−0.193**	**0.0073**	0.039	0.2129	**−0.094**	**0.0484**
CXCL1/KC	−0.014	0.5118	0.042	0.2427	−0.019	0.4335
IL-6	**−0.411**	**<0.0001**	**0.137**	**0.0285**	−0.049	0.2013
MIP-2	0.036	0.2972	**0.133**	**0.0405**	<0.001	0.9737
TNFα	**−0.285**	**0.0014**	0.060	0.1696	−0.021	0.4167
VEGF	−0.065	0.1516	<−0.001	0.9176	**−0.315**	**0.0005**
IL-1β	**−0.280**	**0.0165**	0.068	0.2662	<−0.001	0.9972

Significance and R^2^ values were calculated using a two-tailed Pearson correlation.

IL, interleukin; IT-LPS, intratracheal lipopolysaccharide; LPS; MIP-2, macrophage-inflammatory protein-2; PBS, phosphate buffered saline; RAGE, receptor for advanced glycation end; TNF α, tumor necrosis factors α; VEGF, vascular endothelial growth factor.

## Discussion

Recent studies have used the MouseOx Plus for a variety of reasons including demonstration of hypoxaemia in transgenic mouse models,^17^ differential SaO_2_ following mechanical ventilation^18^ and monitoring of oxygen saturation post toxic gas inhalation.^19^ However, to date, no study has used this system to monitor arterial blood oxygen saturation following IT instillations of LPS, a well-characterised model of ALI/ARDS. This study demonstrated that infrared pulse oximetry can monitor the decline in SaO_2_ following IT-LPS and highlights for the first time the effect of cumulative neutrophil recruitment over several days, which results in oxygen saturation to decrease to 81.1%. A surprising result was that the lowest SaO_2_ levels were observed 96 h post IT-LPS instillation, a time point when markers of pulmonary injury and inflammation were returning to normal. This lag in lung function decline has not been appreciated before but implies that resolution of lung injury worsens oxygenation in mice. The mechanisms for this change are uncertain but may relate to restoration of blood flow to damaged areas of lung resulting in increased ventilation-perfusion mismatch.

An important finding of this study is that SaO_2_ correlates with well-characterised markers of pulmonary injury and inflammation used to assess the extent of ALI/ARDS in mice following IT-LPS. Indeed, SaO_2_ was the only cardiopulmonary parameter measured by the MouseOX Plus that correlated with the specific marker of alveolar epithelial cell damage, BAL RAGE expression. This may suggest that blood oxygen levels can be used in future experiments to verify whether quantitative lung injury markers directly affect lung function. However, a limitation of this study is that arterial blood samples were not analysed directly, in tandem to pulse oximetry to corroborate SaO_2_ readings. Changes in arterial PO_2_ have been shown recently to correlate to SaO_2_.^18^ In addition, our data suggest that SaO_2_ readings continue to drop from 48 to 96 h after IT-LPS even though heart and breath rate remain unchanged at these time points (figure 1C–E). Taken together these data suggest that SaO_2_ monitored during this study is reflective of the relative oxygen saturation within the artery.

Our data also suggest that RAGE expression increased 24 h post IT-LPS even though systemic hypoxaemia as determined by SaO_2_ was unchanged compared to PBS-treated controls. Previous in vitro experiments have suggested that RAGE expression is regulated by hypoxia by HIF1α,^20^ although to date type 1 lung epithelial cells have not been tested. Therefore, these data may reflect hypoxia-independent enzymatic cleavage and/or cytokine-induced RAGE release from epithelial cells or simply an effect of local tissue hypoxia prior to systemic hypoxemia.

Refractory expression of BAL VEGF was observed during the latter stages of lung repair following IT instillation of LPS. VEGF is predominantly expressed by alveolar type II cells in the lung,^21^ with contributions from macrophages and neutrophils during inflammatory responses.^22^ In this context, the role of VEGF in the lung is as a potent stimulus for endothelial and epithelial repair.^23^ ^24^ The decrease in VEGF observed from 96 h in this model closely resembles the reduced VEGF levels observed in patients with ALI, which may be associated with impaired repair responses or reflect specific loss of alveolar type II cells following injury.^25^ Decline in lung function was also maximal at 96 h. This may suggest that future experiments using murine IT-LPS as a model of ALI should monitor this time point in particular. Although we did not extend our time points further than 9 days, it would be interesting in future studies to focus on this phase given that in addition to reduced VEGF levels, BAL neutrophilia and PPI remained elevated compared to resting levels at day 9 post IT-LPS. This was observed even though the levels of inflammatory chemokines associated with leucocyte chemotaxis such as CXCL1/KC and MIP-2 had returned to pre-IT-LPS levels. Taken together, these data are suggestive of permanent damage to alveolar-capillary barriers as a result of IT-LPS. A similar observation can be seen in other experimental models of inflammation when tissue function recovers but restoration of leucocyte populations do not return to predisease levels.^26^

Using pulse oximetry, we found the heart rate of our mice to average around 650–700 bpm (figure 1D). This mirrors recently published data which also used the MouseOx Plus system.^27^ However, resting mice have been previously shown to average 400–600 bpm.^28^ ^29^ Heart rates of this magnitude were most similar to those of mice 24 h post IT-LPS. The MouseOx Plus system we used involved collar clips being placed directly on the animal and allowing them to wander freely in a cage. Therefore, we would suggest this to be a more sensitive reading of cardiac output than older technologies.

As a consequence of lung injury, direct readings obtained by the MouseOx Plus system became easier to monitor (more error-free data points) mainly due to the reduction in the mouse's activity. At extended time points, such as day 9, readings were more challenging not only due to the increased activity of the subject, but also due to regrowth of the hair around the neck and shoulders which can retard the infrared signals. These issues should be considered in future experiments using the MouseOx Plus system.

In conclusion, this study is the first to measure multiple quantitative markers of lung injury and inflammation alongside non-invasive monitoring of cardiopulmonary parameters during a mouse model of ALI. Our data revealed that lung function decline is maximal at 96 h post IT-LPS and that well-characterised indices of lung injury and inflammation correlate with SaO_2_. Pulse oximetry readings are easy to measure, and can be carried out with minimal stress to the animal, providing real-time data indicative of lung function as assessed by SaO_2_. Therefore this parameter may have the potential to predict outcome, help ensure humane endpoints are maintained and reduce animal usage by identifying points at which lung function differs from expected results.
